# Neonatal monocytes exhibit a unique histone modification landscape

**DOI:** 10.1186/s13148-016-0265-7

**Published:** 2016-09-20

**Authors:** Jennifer R. Bermick, Nathalie J. Lambrecht, Aaron D. denDekker, Steven L. Kunkel, Nicholas W. Lukacs, Cory M. Hogaboam, Matthew A. Schaller

**Affiliations:** 1 Department of Pediatrics, Division of Neonatal-Perinatal Medicine, University of Michigan Medical Center, 1540 E. Medical Center Drive, C.S. Mott Children’s Hospital Room 8-621, Ann Arbor, MI 48109 USA; 2 Department of Pathology, University of Michigan Medical Center, Ann Arbor, MI 48109 USA; 3 Department of Medicine, Division of Pulmonary & Critical Care Medicine, Cedars-Sinai Medical Center, Los Angeles, CA 90048 USA

**Keywords:** Histone modification, Epigenetics, Innate immunity, Development, ChIP-sequencing (ChIP-seq)

## Abstract

**Background:**

Neonates have dampened expression of pro-inflammatory cytokines and difficulty clearing pathogens. This makes them uniquely susceptible to infections, but the factors regulating neonatal-specific immune responses are poorly understood. Epigenetics, including histone modifications, can activate or silence gene transcription by modulating chromatin structure and stability without affecting the DNA sequence itself and are potentially modifiable. Histone modifications are known to regulate immune cell differentiation and function in adults but have not been well studied in neonates.

**Results:**

To elucidate the role of histone modifications in neonatal immune function, we performed chromatin immunoprecipitation on mononuclear cells from 45 healthy neonates (gestational ages 23–40 weeks). As gestation approached term, there was increased activating H3K4me3 on the pro-inflammatory *IL1B*, *IL6*, *IL12B*, and *TNF* cytokine promoters (*p* < 0.01) with no change in repressive H3K27me3, suggesting that these promoters in preterm neonates are less open and accessible to transcription factors than in term neonates. Chromatin immunoprecipitation with massively parallel DNA sequencing (ChIP-seq) was then performed to establish the H3K4me3, H3K9me3, H3K27me3, H3K4me1, H3K27ac, and H3K36me3 landscapes in neonatal and adult CD14+ monocytes. As development progressed from neonate to adult, monocytes lost the poised enhancer mark H3K4me1 and gained the activating mark H3K4me3, without a change in additional histone modifications. This decreased H3K4me3 abundance at immunologically important neonatal monocyte gene promoters, including *CCR2*, *CD300C*, *ILF2*, *IL1B*, and *TNF* was associated with reduced gene expression.

**Conclusions:**

These results provide evidence that neonatal immune cells exist in an epigenetic state that is distinctly different from adults and that this state contributes to neonatal-specific immune responses that leaves them particularly vulnerable to infections.

**Electronic supplementary material:**

The online version of this article (doi:10.1186/s13148-016-0265-7) contains supplementary material, which is available to authorized users.

## Background

Neonates are uniquely susceptible to infections, with preterm infants (born before 37 weeks of gestation) being the most vulnerable [1]. Up to 25 % of extremely preterm infants develop a culture-positive bacterial infection during their initial hospitalization, which increases their risk of death and long-term neurodevelopmental impairment [2]. Neonatal susceptibility to infection is thought to be due to the need for invasive but life-saving medical interventions, immaturity of the immune system, and disordered regulation of inflammation [3, 4].

Protection against infection occurs through the combined efforts of the innate and adaptive immune systems. Monocytes, which mature into macrophages in the tissues, are part of the innate immune system and act as the first line of defense, sensing pathogens and presenting them to cells of the adaptive immune system. Type 1 macrophages express pro-inflammatory cytokines, exhibit strong phagocytic activity, and contribute to the acute inflammatory response, resulting in clearance of pathogens. In contrast, type 2 macrophages express anti-inflammatory cytokines, contribute more to the maintenance of chronic inflammation and tissue fibrosis, and are less efficient at clearing pathogens [5]. There is a predominance of type 2 cytokine expression during most normal pregnancies, thought to suppress cellular immunity and protect the fetus from abortive responses [6]. This type 2 cytokine predominance continues in the newborn for several weeks after delivery and in the preterm infant may be prolonged [7]. In neonates, this type 2 skew results in dampened production of the pro-inflammatory cytokines IL-1β, IL-6, IL-12, and TNF-α, impacting their ability to fight infections and clear pathogens [8–12]. Mechanisms controlling developmentally related innate immune system phenotype differences are poorly understood.

Epigenetics studies how genetic and environmental factors interact to alter DNA structure without affecting the underlying genetic code. Epigenetic modifications include DNA methylation, histone modifications, and microRNA expression. These modifications collectively activate or silence gene transcription by influencing chromatin structure and stability or by altering how DNA interacts with transcription factors. The amino acid that is most often modified in histones is lysine, and its position in the histone tail can determine if the added modification allows an open or closed configuration of the gene. The addition of three methyl groups on lysine (K) 4 of histone (H) 3 (i.e., H3K4me3) leads to activation of gene transcription. H3K9me3 and H3K27me3 are both associated with gene silencing as they promote chromatin compaction and make gene promoters inaccessible to transcription factors [13, 14]. H3K4me1 indicates poised enhancers while acetylation of H3K27 (i.e., H3K27ac) marks active enhancers, both of which serve as distal regulators of gene expression [15, 16]. H3K36me3 is associated with either activation or inhibition of gene transcription based on surrounding marks [17].

Histone modifications have been found to underlie normal developmental changes in the immune system, contributing to cell lineage decisions and influencing how cells respond to external stimuli [15, 18]. There has been increasing interest in developmental-specific epigenetic events that may underlie the unique responses of the neonatal immune system, and some work has evaluated how DNA methylation and microRNA expression impact these responses in term neonates [19, 20]. Our work is the first to investigate the role of histone modifications in the development and maturation of the neonatal immune system. We demonstrate that in monocytes, a decrease in the poised enhancer mark H3K4me1 parallels an increase in the active promoter mark H3K4me3 as development progresses from neonate to adult. We also show that a lack of H3K4me3 in neonatal monocytes is associated with a reduction in gene expression in vital immunologic pathways. These findings suggest that the unique histone modification landscape in neonatal monocytes may be contributing to the heightened infection risk present in the neonatal period.

## Results

### H3K4me3 increases at mononuclear cell cytokine promoters as development progresses

**Fig. 1:**
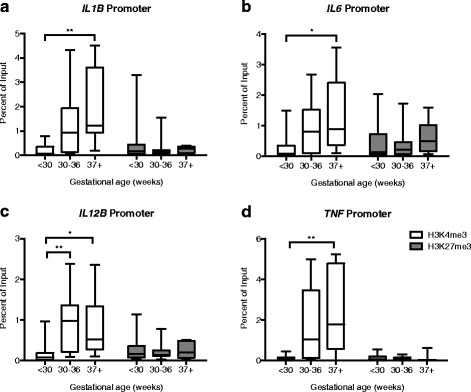
The activating histone modification H3K4me3 increases on pro-inflammatory cytokine promoters as development progresses over the third trimester. ChIP assays were performed to determine the H3K4me3 and H3K27me3 profiles throughout fetal development of the **a** *IL1B* promoter, **b** *IL6* promoter, **c** *IL12B* promoter, and **d** *TNF* promoter. <30 *n* = 25, 30–36 *n* = 10, 37+ *n* = 10. *p < 0.05, **p < 0.01. *Box* represents 25th–75th percentile range, and *whiskers* represent minimum to maximum values

To determine if histone modifications contribute to the attenuated pro-inflammatory responses observed in preterm neonates, we performed a chromatin immunoprecipitation assay (ChIP) using anti-H3K4me3 and anti-H3K27me3 antibodies to evaluate the promoter regions of the pro-inflammatory cytokine genes *IL1B* (Fig. 1a), *IL6* (Fig. 1b), *IL12B* (Fig. 1c), and *TNF* (Fig. 1d). The activating histone modification H3K4me3 was significantly increased on the promoters of all four cytokines in term infants, compared to extremely preterm infants (Fig. 1). There was no change in the repressive histone modification H3K27me3 over development on any of the promoters analyzed (Fig. 1). These findings suggest that the *IL1B*, *IL6*, *IL12B*, and *TNF* promoters of the preterm innate immune system are less accessible to transcription factors than in term neonates, which may contribute to the attenuated pro-inflammatory responses exhibited by preterm infants.

### The abundance and location of monocyte H3K4me3 changes throughout development

**Fig. 2:**
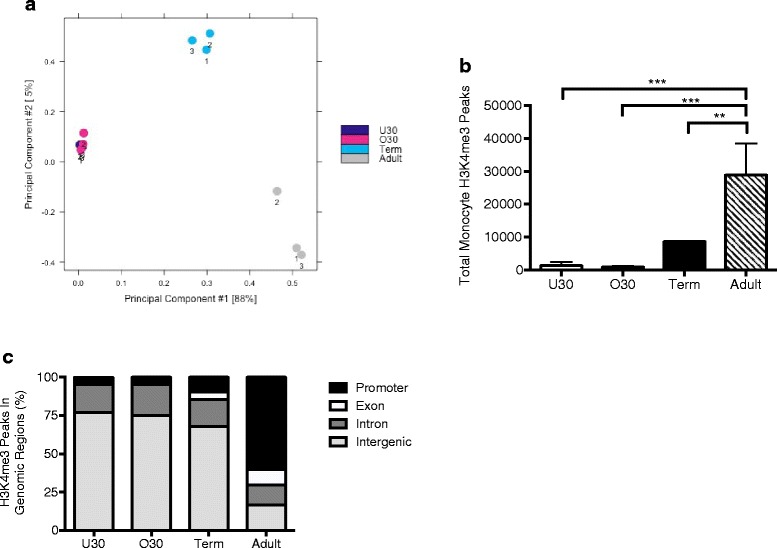
The number and location of H3K4me3 peaks throughout the monocyte genome change over the course of development. **a** Principle component analysis showing clustering of experimental groups based on H3K4me3-binding sites and affinity for those sites. 1, 2, 3 = replicate number. **b** H3K4me3 total peaks in the monocyte genome by age. *Box* represents mean, and *bar* represents SD. **p < 0.01, ***p < 0.001. **c** Location of H3K4me3 peaks in monocytes by age

To determine the overall profile of H3K4me3 in innate immune cells throughout development, we performed chromatin immunoprecipitation with massively parallel DNA sequencing (ChIP-seq), allowing us to identify all H3K4me3 sites in the monocyte genome. We evaluated the global distribution of H3K4me3 in CD14+ monocytes from four experimental groups: under 30-week preterm infants (U30), over 30-week preterm infants (O30), term infants (Term), and healthy adults (Adult). Figure 2 summarizes the differences in H3K4me3 deposition in monocytes among different developmental stages. A principle component analysis of the H3K4me3 monocyte peak locations and affinity for these locations shows that the preterm monocytes cluster together, the term monocytes cluster together, and the adult monocytes cluster together, but these three groups are distinct from each other both in location and abundance of H3K4me3 (Fig. 2a). A closer look at the total amount of H3K4me3 in the monocyte genome shows that there is an increase in total number of H3K4me3 peaks as development progresses toward term, although the term neonates still have significantly less H3K4me3 than the adults (Fig. 2b). To obtain a broad view of H3K4me3 distribution, we divided the human genome into four distinct categories according to the UCSC Genome Browser known genes: promoters (1 kb upstream or downstream from transcriptional start sites (TSSs)), exons, introns, and intergenic regions [21, 22]. The majority of H3K4me3 peaks in the preterm monocytes were located in introns and intergenic regions, with less than 5 % of the peaks associated with promoters and exons. There were slightly more H3K4me3 peaks associated with promoter and exon sites in the term monocytes, ~15 %, although the majority were still located in noncoding regions of the genome. In contrast, the adult monocytes had the majority of their H3K4me3 peaks located at promoters and exons (70 %), with only a minority in other regions of the genome (Fig. 2c). Over 75 % of the intergenic H3K4me3 peaks present in the neonatal monocytes were also present in the adult monocytes, suggesting that the intergenic neonatal peaks remained stable as development progressed and that the majority of new H3K4me3 peaks acquired were in promoter and exon locations. The total number of peaks in each location is detailed in Additional file 1: Table S1.

### H3K4me3 is associated with distinctly different gene ontology pathways in neonatal and adult monocytes

**Fig. 3:**
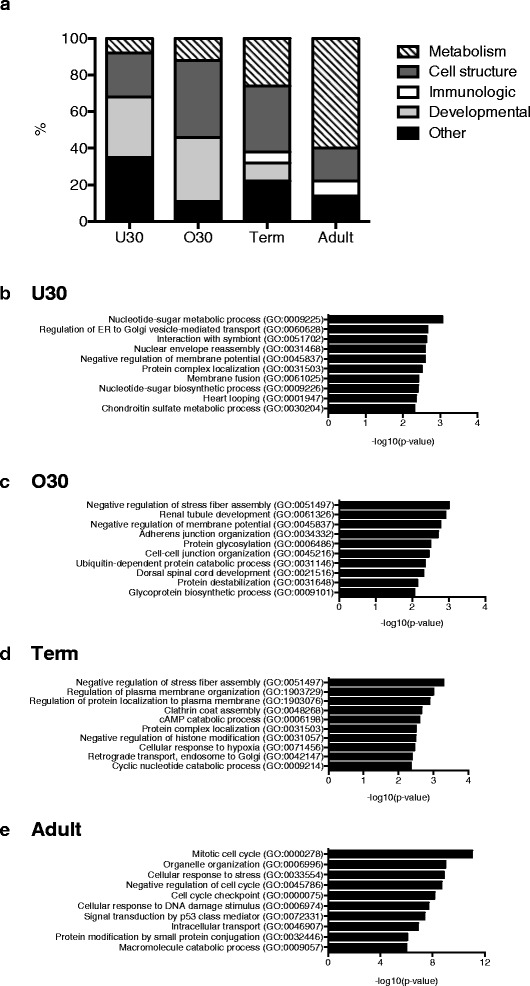
Gene pathways enriched for H3K4me3 evolve throughout development and into adulthood. **a** Broad biological gene ontology pathways associated with H3K4me3 monocyte peaks by age. The top non-synonymous biological gene ontology pathways associated with H3K4me3 peaks in **b** under 30 week preterm monocytes, **c** over 30 week preterm monocytes, **d** term monocytes, and **e** adult monocytes

A large proportion of H3K4me3 peaks in the monocytes of all age groups were associated with gene pathways required for cell survival and basic function including metabolism, cell structure, cell signaling, and intracellular transport (Fig. 3a). The major gene ontology pathways that changed over the course of maturation included developmentally related pathways and immunologically important pathways (Fig. 3a). The preterm monocytes had 30–35 % of their H3K4me3 peaks associated with developmentally important pathways, including pathways involved in spinal cord, heart, and renal development. The term monocytes had approximately 10 % of their H3K4me3 peaks associated with developmentally important pathways, while the adult monocytes had no peaks associated with these pathways. Conversely, the U30 monocytes had no H3K4me3 peaks associated with immunologically important pathways, including responses to pathogens, cytokine and chemokine production, and antigen presentation. The O30 monocytes had less than 1 % (4 total) of H3K4me3 peaks associated with immunologically important pathways, while the term monocytes had approximately 6 % (30 total) and the adult monocytes had around 8 % (57 total). The immunologically important pathways enriched for H3K4me3 are detailed in Additional file 1: Table S2, and the top ranking gene ontology biological pathways enriched for H3K4me3 by age group are shown in Fig. 3b–e. Of note, interferon responsive genes, including *IFNB1*, *CXCL10*, and *ISG15*, did not have differentially bound H3K4me3 between neonatal and adult monocytes.

There were numerous microRNA (miR) promoters with increased H3K4me3 peaks in the adult monocytes. miR-21 is the lone miR identified with known immune function and is involved in the negative regulation of TLR4 signaling [23]. All miRs with differential binding between adult and neonatal monocytes are listed in Additional file 1: Table S3. Immune cell activation requires a metabolic shift from oxidative phosphorylation to glycolysis or lipid metabolism to account for the up-regulation of a large number of inflammatory genes, and neonatal immune cells have difficulty undergoing this metabolic shift effectively [24–26]. As a large proportion of H3K4me3 peaks gained over the course of development are in metabolic pathways, we evaluated if differentially bound H3K4me3 peaks between term neonatal and adult monocytes could account for the differences in their ability to undergo this critical metabolic shift. We found multiple genes crucial to glycolysis and lipid metabolism with increased H3K4me3 binding in adult monocytes (Additional file 1: Table S4). The gene ontology pathways associated with H3K4me3 peaks present only in the adult monocytes are outlined in Additional file 2: Figure S1.

### A decline in monocyte H3K4me1 parallels an increase in H3K4me3 during development

**Fig. 4:**
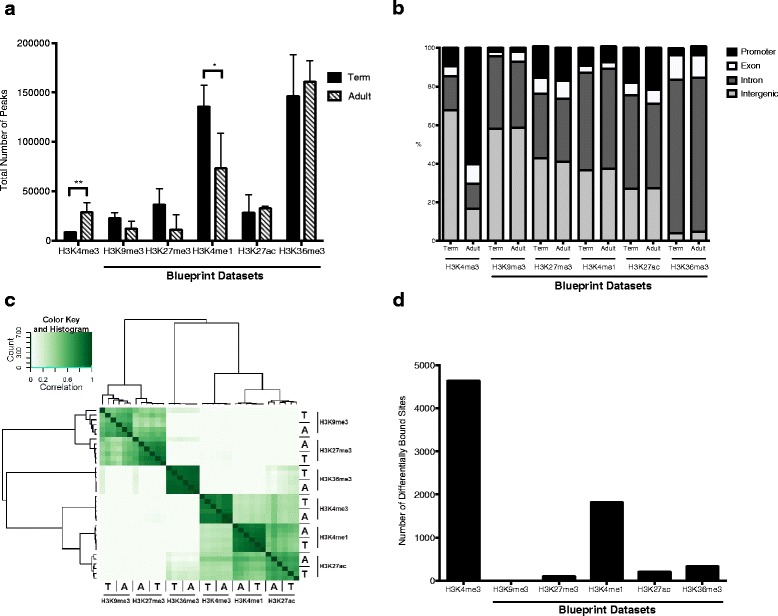
Monocytes display a decrease in the enhancer mark H3K4me1 with a parallel increase in the activating mark H3K4me3 as development progresses from neonate to adult. **a** Total monocyte peak numbers of the histone modifications H3K4me3 (from our dataset), H3K9me3, H3K27me3, H3K4me1, H3K27ac, and H3K36me3 (from the Blueprint Consortium) in term neonatal and adult CD14+ monocytes. *p < 0.05, **p < 0.01. **b** Genomic location of histone modification peaks in term neonatal and adult monocytes. **c** Correlation heatmap comparing histone modification peak occupancy sites. *T* = term monocytes, *A* = adult monocytes. **d** Number of differentially bound histone modification peaks between neonatal and adult monocytes

To evaluate if histone modifications other than H3K4me3 change significantly over the course of development, we took advantage of ChIP-seq datasets from the Blueprint project (large scale research project generating reference epigenomes for mechanistic studies of health and disease). The Blueprint project has performed ChIP-seq experiments targeting common histone modifications in both adult monocytes and monocytes from term neonatal umbilical cord blood. These experiments include immunoprecipitation of the suppressive marks H3K9me3 and H3K27me3, the enhancer marks H3K4me1 and H3K27ac, and the more versatile mark H3K36me3, which can serve as both an activating and repressive mark. Each Blueprint experiment contains datasets for these histone marks in umbilical cord blood CD14+ monocytes from three healthy term neonates and peripheral blood CD14+ monocytes from three healthy adults. Information obtained from each of these datasets was processed in the same fashion as the H3K4me3 monocyte dataset described above. The H3K4me3 dataset used for comparison is from the neonatal and adult samples that we acquired and processed, not from the Blueprint Consortium. Adult monocytes had significantly more H3K4me3 than neonatal monocytes, but less H3K4me1 (Fig. 4a). Both the neonatal and adult monocytes had comparable amounts of H3K9me3, H3K27me3, H3K27ac, and H3K36me3 peaks (Fig. 4a). The broad genomic locations of the H3K9me3, H3K27me3, H3K4me1, H3K27ac, and H3K36me3 marks were not significantly different between the neonatal and adult monocytes (Fig. 4b). A closer analysis of the location of these marks in relation to each other demonstrated that the repressive marks H3K9me3 and H3K27me3 shared similar locations and the activating/enhancing marks H3K4me3, H3K4me1 and H3K27ac shared similar locations, while H3K36me3 had significantly different locations than the other marks (Fig. 4c). The histone modifications with the largest number of differentially bound peaks between neonatal and adult monocytes were H3K4me3 and H3K4me1 (Fig. 4d). There were very few bivalent domains, containing H3K4me3 and H3K27me3 or H3K9me3, in the neonatal and adult monocytes (Additional file 3: Figure S2). These results demonstrate that monocytes lose the enhancer mark H3K4me1 and gain the activating mark H3K4me3 as development progresses from neonate to adult. Other histone modifications stay relatively constant in both number and location.

**Fig. 5:**
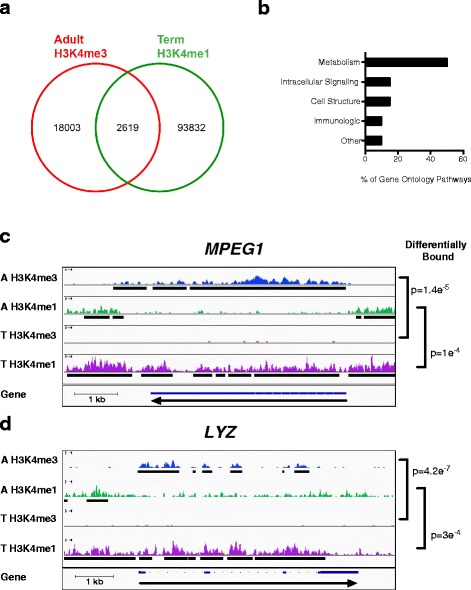
A subset of H3K4me1 in neonatal monocytes appears to directly convert to H3K4me3 in adult monocytes. **a** Venn diagram showing that approximately 12 % of H3K4me3 peaks differentially bound between neonatal and adult monocytes have differentially bound H3K4me1 peaks at the same location. **b** Broad biological gene ontology pathways associated with shared adult H3K4me3 and term neonatal H3K4me1 peaks. **c** *MPEG1* and **d** *LYZ* have significantly increased H3K4me1 across the whole gene in neonatal monocytes without appreciable H3K4me3, which seems to convert to H3K4me3 at identical locations in adult monocytes with a parallel decrease in H3K4me1. *A* = adult, *T* = term. H3K4me3 peaks display the read coverage for H3K4me3 from one of the representative replicates from each group. *Black bars* display the H3K4me3 consensus peaks after only peaks present in at least two replicates are combined and analyzed. *MPEG1* macrophage expressed 1, *LYZ* lysozyme

Given the shift from high H3K4me1/low H3K4me3 in neonatal monocytes to low H3K4me1/high H3K4me3 in adult monocytes, we decided to more closely analyze the location of these marks to determine if some of the neonatal H3K4me1 marks were being directly converted to H3K4me3 in the adult monocytes or if the additional H3K4me3 marks in the adults were being placed de novo. Sites with increased H3K4me3-binding affinity in adult monocytes were compared to sites with increased H3K4me1-binding affinity in neonatal monocytes, and significant overlap was noted (Fig. 5a). Approximately 12 % of the differentially bound H3K4me3 sites in adult monocytes also contained differentially bound H3K4me1 in neonatal monocytes. The gene ontology pathways associated with these overlapping marks are detailed in Fig. 5b. A closer look at some of these genes revealed that the increased H3K4me1 in the neonatal monocytes shared an identical location with the increased H3K4me3 in the adult monocytes, supporting the hypothesis that a direct conversion between H3K4me1 and H3K4me3 is occurring at some genes during monocyte development (Fig. 5c, d).

### The presence and abundance of H3K4me3 in monocytes influences immune function

**Fig. 6:**
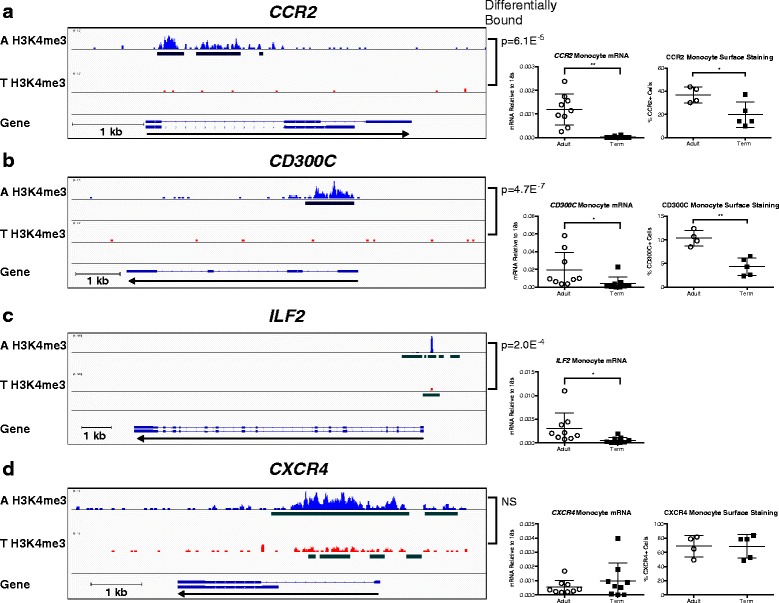
Differential binding of H3K4me3 at gene promoter sites during monocyte development results in changes in gene and protein expression. **a** The presence of H3K4me3 peaks around the promoter site of *CCR2* in adult monocytes without an appreciable H3K4me3 peak in term neonatal monocytes is associated with significantly more *CCR2* mRNA expression and CCR2 surface staining by flow cytometry in unstimulated adult monocytes. **b** The presence of H3K4me3 peaks around the promoter site of *CD300C* in adult monocytes without an appreciable H3K4me3 peak in term neonatal monocytes is associated with significantly more *CD300C* mRNA expression and CD300C surface staining by flow cytometry in adult monocytes only after LPS 100 ng/mL in vitro stimulation. **c** Increased H3K4me3 peaks around the promoter site of *ILF2* in adult monocytes are associated with significantly more *ILF2* mRNA expression in unstimulated adult monocytes. **d** Comparable amounts of H3K4me3 peaks around the promoter site of *CXCR4* in adult and term neonatal monocytes are associated with comparable *CXCR4* mRNA expression and CXCR4 surface staining by flow cytometry in unstimulated monocytes. *A* = adult, *T* = term. H3K4me3 peaks display the read coverage for H3K4me3 from one of the representative replicates from each group. *Black bars* display the H3K4me3 consensus peaks after only peaks present in at least two replicates are combined and analyzed. For the mRNA studies, adult *n* = 9, term *n* = 9. For the flow cytometry studies, adult *n* = 4, term *n* = 5. *p < 0.05, **p < 0.01

**Fig. 7:**
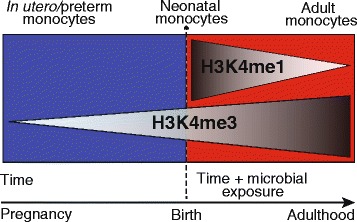
Proposed scheme of the evolution of the monocyte histone modification landscape over the course of development from preterm neonate to adult

Given the association of H3K4me3 with promoter and 5′ coding regions of actively transcribed genes, we evaluated immunologically important monocyte genes with H3K4me3 peaks surrounding their promoter regions [27, 28]. The genes evaluated did not have other differentially bound histone modification peaks within 2 kb of their TSSs. As preterm monocytes had very few H3K4me3 peaks associated with promoter regions, we focused solely on the function of term neonatal and adult monocytes. Neonatal umbilical cord blood CD14+ monocytes and adult peripheral blood CD14+ monocytes were cultured in vitro with LPS 100 ng/mL (TLR4 ligand, simulates gram-negative bacterial infection) or polyI:C 25 μg/mL (TLR3 ligand, simulates viral infection), and messenger RNA (mRNA) expression was measured before culture (unstimulated) or 2 h after stimulation and receptor surface staining was measured before culture (unstimulated) or 6 h after stimulation. Neither the neonatal nor adult monocytes demonstrated significant up-regulation of inflammatory gene expression upon polyI:C stimulation, with no difference observed between the neonatal and adult monocyte responses to TLR3 activation. Therefore, the analysis of monocyte function included LPS stimulation only. The evaluated genes were categorized by the amount of bound H3K4me3 shared between adult and neonatal monocytes and whether gene expression was present at baseline or required LPS stimulation to be expressed. The first category of genes had H3K4me3 peaks surrounding promoters in the adult monocytes without any appreciable peaks in the term neonatal monocytes. A subset of these genes had increased gene expression and surface receptor expression at baseline in the adult monocytes (Fig. 6a, *CCR2* as example), while another subset had increased gene expression and surface receptor expression in the adult monocytes only after LPS stimulation (Fig. 6b, *CD300C* as example). The second category of genes had H3K4me3 peaks surrounding promoters in both the adult and term neonatal monocytes but had differential binding with increased binding amount and affinity in the adult monocytes. These genes had increased gene expression in the adult monocytes either at baseline or with LPS stimulation depending on the function of the gene product (Fig. 6c, *ILF2* as example). The third category of genes had comparable H3K4me3 binding near promoters in both adult and term neonatal monocytes with equivalent gene expression and surface receptor expression both at baseline and upon LPS stimulation (Fig. 6d, *CXCR4* as example). Representative flow cytometry plots for these surface receptors are shown in Additional file 4: Figure S3. The top 50 genes with differentially bound H3K4me3 near their promoter sites can be found in Additional file 1: Table S5. Consistent with our conventional ChIP data, there was increased H3K4me3 binding at the promoter sites of *IL1B* and *TNF* with increased mRNA gene expression and secreted protein levels upon LPS stimulation (Additional file 5: Figure S4). These results suggest that both the presence and abundance of H3K4me3 at gene promoters are critical to promoting active gene expression, rather than peak presence alone. Figure 7 summarizes the main findings of this study.

## Discussion

Around the time of birth, the immune system has to transition from a sterile intrauterine environment to an environment full of foreign antigens and microbes. During this transition, the neonatal immune system must avoid excessive pro-inflammatory responses so that rapid colonization of the skin and gastrointestinal tract with beneficial microbial organisms can occur [29, 30]. Disruption in normal neonatal microbial colonization can have long-lasting deleterious effects and is thought to contribute to the development of allergic diseases, including asthma [31, 32]. The fetal and neonatal immune system polarization toward immunomodulatory type 2 cytokine responses rather than more adult-like pro-inflammatory type 1 cytokine responses not only aids in this transition but also leaves neonates highly susceptible to infections [3, 7]. The factors controlling the developmental progression of immature neonatal immune responses to mature adult immune responses are poorly understood, leaving a large deficit in our ability to prevent, diagnose, and effectively treat neonatal infections. The present study strongly suggests that alterations in H3K4 methylation contribute to neonatal innate immune deficits, especially noteworthy in preterm infants.

Our study is the first to evaluate the contribution of histone modifications to the neonatal immune response and demonstrates that monocytes have a developmentally regulated loss of the poised enhancer mark H3K4me1 with a concurrent gain of the activating mark H3K4me3. Other histone modifications including H3K9me3, H3K27me3, H3K27ac, and H3K36me3 stay relatively constant in both number and location throughout development and are unlikely to make a significant contribution to developmental specific changes in monocyte function. The poised enhancer mark H3K4me1 and the active promoter mark H3K4me3 do not co-localize, with genes either demonstrating high H3K4me1 and low H3K4me3 or the converse. This may reflect the conversion of H3K4me1 to H3K4me3 through the intermediate H3K4me2 rather than a complete loss or gain of histone methylation at these sites [33, 34]. In agreement with this, we have identified a subset of genes in which H3K4me1 appears to be directly converted to H3K4me3 as development progresses with genes moving from a “poised” to an active configuration. This subset of genes only comprises a small amount (12 %) of the H3K4me3 gained in monocytes as development progresses, suggesting that the majority (88 %) of H3K4me3 is deposited de novo.

H3K4me3 is typically located at promoter regions of actively transcribed genes [22, 27], so it is interesting that the majority of H3K4me3 in neonatal monocytes is in noncoding regions of the genome, including introns and intergenic regions. It is unclear what role H3K4me3 is playing in these locations, but it may be serving a distal regulatory role that has not previously been described. Most of the neonatal H3K4me3 intergenic peaks are also present in the adult monocytes, so it appears that the majority of the new H3K4me3 peaks gained throughout development are being deposited in promoter and exon locations. Distinct differences also exist in gene ontology pathways associated with H3K4me3 in neonatal and adult monocytes. Neonatal monocytes have numerous H3K4me3 peaks associated with developmentally related pathways, but these peaks are not present in adult monocytes. The neonatal monocytes also have significantly less metabolic pathways associated with H3K4me3, which is important because a metabolic shift from oxidative phosphorylation to either glycolysis or lipid metabolism is required to accommodate the induction of a large number of genes upon immune cell activation [24, 25]. Recent evidence suggests that neonatal immune cells lack the ability to perform this metabolic shift [26]. We identified multiple genes crucial to glycolysis and lipid metabolism containing H3K4me3 peaks in adult monocytes with decreased or absent H3K4me3 peaks in the neonatal monocytes. In light of this, we believe that H3K4me3 may be an important factor regulating the ability of monocytes to undergo this critical metabolic shift, and a lack of this mark in metabolic pathways in neonatal monocytes may contribute to their dampened inflammatory response. In addition, adult monocytes have significantly more H3K4me3 peaks associated with immunologically important pathways than neonatal monocytes, and the expression of some genes vital to the monocyte inflammatory response to pathogens are decreased in neonates due to either a complete absence of or decreased abundance of H3K4me3 at their promoters. There are also numerous miR promoters with increased H3K4me3 in adult monocytes, suggesting that the ability to perform nuanced regulation of complex pathways in monocytes is not fully developed until adulthood. The increased H3K4me3 gained by monocytes during development appears to “mature” the immune system, resulting in more robust and appropriate inflammatory responses and optimal clearance of pathogens. Taken together, these results suggest that neonatal immune cells exist in an epigenetic state that is distinctly different from adult immune cells and that this epigenetic state is a major contributor to neonatal specific immune responses.

The increase in H3K4me3 deposition that occurs between extremely preterm and term infants appears to be triggered solely by stage of development, as these monocytes have yet to be exposed to the ex-utero environment. This is consistent with what occurs during gamete formation and early embryo development, when conserved post-translational histone modification additions and removals allow reprogramming of the epigenetic landscape during a critical developmental window [35]. However, the increase in total H3K4me3 deposition between term neonatal to adult monocyte likely results from a combination of developmental-specific and environmental exposure-related triggers. Stimulation of immune cells by pathogens or cytokines can prompt the deposition or removal of H3K4me3 at functionally important genes and is critical for the establishment and maintenance of macrophage and T cell phenotypes [36–39]. Other environmental exposures can also alter histone modification patterns, including high-fat diet, diabetes-associated hyperglycemia, and environmental pollutants and toxins [40–43]. These exposure-induced epigenetic alterations have also been shown to occur at the level of bone marrow progenitor cells and can be inherited by daughter cells, resulting in permanent and heritable changes in histone modification patterns in hematopoietic cells [41]. We postulate that the increased H3K4me3 deposition seen in monocytes over the course of postnatal development is prompted by exposure to the ex-utero environment. We believe these environmental stimuli prompt the appropriate methyltransferase(s) to deposit H3K4me3 at and around gene promoter sites to remodel the epigenetic landscape of the monocytes to a more mature and functional state.

There are some limitations to this study. We attempted to control for expected variability in primary human monocytes by using pooled neonatal samples and three replicates in our ChIP-seq experiments, but there may have been some sex-specific or genetic variability in H3K4me3 patterns that were masked by this approach. Additionally, while we evaluated a broad number of histone modifications throughout monocyte development, there are likely other factors that influence developmental-specific changes in monocyte function, including other histone modifications (H3K79me2, H3K9ac, H4K20me1), DNA methylation, miR expression, and non-epigenetic factors that were not included in our study.

## Conclusions

Our study reveals an increase in monocyte H3K4me3 deposition at promoter sites of immunologically and metabolically important genes as development progresses from neonate to adult, which correlates with the ability to mount a more robust inflammatory response. These results show that neonatal monocytes have a unique histone modification landscape that contributes to their attenuated response to pathogens and contributes to the neonate’s vulnerability to infection. Further studies are needed to determine the factors regulating this developmental change in monocyte H3K4me3 amount and location and whether these factors can be targeted to improve neonatal outcomes before, during, and after infection.

## Methods

### Participants

The research protocol was approved by the local Institutional Review Board, and written informed consent was obtained from adult participants and parents of all neonatal participants. We prospectively enrolled term (gestational age >37 weeks), late preterm (gestational age 30–36 weeks), and extremely preterm (gestational age <30 weeks) infants born at the University of Michigan Medical Center, between May 2012 and August 2015. Exclusion criteria included the presence of histologic chorioamnionitis. We enrolled a total of 16 adults and 34 extremely preterm, 19 late preterm, and 33 term infants.

### Blood

Peripheral blood was collected from participating healthy adults. Umbilical cord blood was collected from participating infants immediately after the delivery of the placenta. The blood was sent to the University of Michigan blood bank and was stored at 4 °C. The umbilical cord blood samples were retrieved from the blood bank and were processed anywhere from day of life 1 through day of life 33, based on when parental informed consent was obtained. The day of sample collection and processing had no effect on the histone methylation of any of the promoters studied. Cord blood samples used to measure mRNA expression were collected no later than day of life 5. Diluted blood (1:2 with sterile 0.9 % saline) was used to harvest umbilical cord mononuclear cells by Ficoll-Isopaque density gradient centrifugation. Mononuclear cells were used for chromatin immunoprecipitation. Mononuclear cells were also subjected to CD14+ magnetic cell isolation according to the manufacturer’s instructions (Miltenyi Biotec). On average, the purity of the monocytes was greater than 90 % by CD14 flow cytometry. Isolated CD14+ cells were aliquoted into cryovials in recovery cell culture freezing media (Life Technologies) and were stored at −80 °C prior to use.

### Chromatin immunoprecipitation (ChIP)

The total cells used for ChIP ranged from 8.0 × 10^5^–4.1 × 10^6^ per sample. Cells were fixed for 10 min at 37 °C in 18.5 % paraformaldehyde. Glycine ×10 was added to quench extra paraformaldehyde. Cells were washed on ice with ice-cold PBS, were lysed in SDS lysis buffer (1 % SDS, 10 mM EDTA, 50 mM Tris-HCl), and underwent syringe passage three times with a 27-gauge needle. The DNA was sheared by ultrasonication for 3 × 10 s pulses at 50 % amplitude (Branson Digital Sonifier 450). The lysates were cleared by centrifugation and were diluted in ChIP dilution buffer (0.01 % SDS, 1.1 % Triton X-100, 2 mM EDTA, 20 mM Tris-HCl, 150 mM NaCl). A sample of “input DNA” was collected, totaling 5 % of the total chromatin, and was stored at 4 °C. Protein-DNA complexes were immunoprecipitated with 4 μg of the following antibodies overnight at 4 °C with rotation: IgG (Millipore, PP64B), H3K4me3 (Abcam, ab8580), and H3K27me3 (Active Motif, 39155). Antibody-protein-DNA complexes were captured using salmon sperm DNA/protein A-agarose beads for 1 h at 4 °C with rotation. The bead complexes were washed with low-salt immune complex buffer (0.1 % SDS, 1 % Triton X-100, 2 mM EDTA, 20 mM Tris-HCl, 150 mM NaCl), high-salt immune complex buffer (0.1 % SDS, 1 % Triton X-100, 2 mM EDTA, 20 mM Tris-HCl, 500 mM NaCl), LiCl immune complex buffer (0.25 M LiCl, 1 % NP40, 1 % deoxycholate, 1 mM EDTA, 10 mM Tris-HCl), and TE buffer (10 mM Tris-HCl, 1 mM EDTA). The protein-DNA complexes and the “input DNA” were then eluted using a 1 % SDS, 0.1 M NaHCO_3_ buffer and were disrupted by heating at 65 °C for 5–24 h. DNA was extracted using phenol/chloroform and ethanol precipitation. Real-time PCR was conducted with the ABI Prism 7700 Sequence Detection System using the SYBR Green PCR reagent (Applied Biosystems) following the manufacturer’s instructions. Dissociation curve analysis was performed for all primers to ensure a single product with the expected melting curve characteristics. PCR was conducted using promoter-specific ChIP primers (Additional file 1: Table S6). Percent of input was calculated as follows: %(ChIP/Total input) = 2^[(Ct_input_ − Log{20,2}) − Ct_ChIP_] × 3 × 100 %.

### Cell culture

Cryovials of CD14+ cells were thawed, and cells were washed twice in sterile RPMI. Cells were then plated in 96-well polystyrene culture plates at 5 × 10^4^ cells/well in 100 μl of RPMI medium containing 400 mM/L l-glutamine, 10 % adult human serum, 1 % penicillin/streptamycin, 1 % sodium pyruvate, and 1 % nonessential amino acids. The cells were stimulated with 100 ng/mL lipopolysaccharide (Sigma, purified from E. coli 055:B5) or 25 μg/mL polyI:C (Invivogen) and were incubated at 37 °C and 5 % CO_2_. Cells were collected for mRNA processing 0 and 2 h after stimulation, and supernatant was collected for protein analysis 24 h after stimulation. Cells were collected for flow cytometry 0 and 6 h after stimulation.

### mRNA and protein measurement

At the time of cell collection, the cells were separated from the supernatant by centrifugation at 5000 RPM for 5 min at 4 °C. Cellular mRNA was then extracted and purified using an RNeasy Micro Kit (Qiagen) according to the manufacturer’s instructions. Complementary DNA was synthesized using murine leukemia virus reverse transcriptase (Applied Biosystems) and incubated at 37 °C for 90 min, followed by 95 °C for 5 min to stop the reaction. Real-time quantitative PCR was multiplexed using Taqman primers with a FAM-conjugated probe and 18 s with a VIC-conjugated probe (Applied Biosystems) to measure transcription of *CCR2*, *CD300C*, *ILF2*, *CXCR4*, *IL1B*, and *TNF*. Resulting mRNA levels were normalized to the housekeeping gene 18s and compared using the ΔCT method. All reactions were run on an ABI Prism 7700 Sequence Detection System using Universal PCR reagent (Applied Biosystems). The protein levels of the cytokines IL-1β and TNF were measured from cell culture supernatants by Bioplex assay (Bio-Rad).

### Flow cytometry

CD14+ monocytes were resuspended in PBS with 1 % FCS and 0.002 M EDTA, and Fc receptors were blocked with purified human IgG. Surface markers were identified using antibodies (clones) against the following antigens, all from BioLegend: CD14 (M5E2), CCR2 (K036C2), CD300C (TX45), and CXCR4 (12G5) each at a 1:100 dilution. Cells were labeled according to the manufacturer’s instructions. Flow cytometry was performed on a NovoCyte machine with 405, 488, and 640 nm lasers.

### High throughput sequencing

Purified umbilical cord blood CD14+ monocytes (including both CD16− and CD16+ subsets) of extremely preterm infants (<30 weeks gestation), late preterm infants (30–36 weeks gestation), term infants (37+ weeks gestation), and healthy adults were used for ChIP-seq. Each age group contained three biological replicates. To obtain approximately 1 × 10^6^ cells per immunoprecipitation, each neonatal sample replicate contained three pooled samples (Additional file 1: Table S7). ChIP was performed as described above, with modification of the following ultrasonication settings: 40 % amplitude, 0.7 s ‘on’, and 1.3 s ‘off’ for 240 s on wet ice. The immunoprecipitation was performed with 4 μg of the H3K4me3 antibody (Abcam, ab8580). DNA from each ChIP experimental group was treated with 30 μg RNase-A (Qiagen) and was incubated at 37° C with intermittent shaking for 1 h. The samples were purified using a MinElute PCR purification kit (Qiagen) following the manufacturer’s instructions. End repair of the sonicated DNA was performed by incubating the samples with a kinase mix (Additional file 1: Table S8) and incubating at 37 °C for 60 min, followed by incubation at room temperature for 10 min. The samples were then incubated with a blunting mix (Additional file 1: Table S9) at 16 °C for 10 min, followed by 12 °C for 20 min, then incubation on ice for 30 min. The samples were then purified using a MinElute PCR purification kit (Qiagen) following the manufacturer’s instructions. 3′ dA-tailing of the blunt DNA was performed by adding a dA-tailing mix (Additional file 1: Table S10) to each sample and incubating at 30 °C for 60 min. The samples were then purified using a MinElute PCR purification kit (Qiagen) following the manufacturer’s instructions. Oligonucleotide adapters and PCR primers are shown in Additional file 1: Table S11. The universal forward PCR primer was reconstituted to 25 μM in TE buffer, and each of the reverse PCR primers was reconstituted to 10 μM in TE buffer and frozen at −80^o^C until use. Adapters A and B were combined and reconstituted in 5X Crimson-Taq Buffer (New England BioLabs) at a concentration of 5 μM each and were frozen at −80 °C until use. After thawing, the A/B adapter mix was incubated on a PCR machine with the following settings: 25 °C for 2 min, 95 °C for 2 min, 72 °C for 1 min, 0.1 °C/s to 30 °C, 30 °C for 5 min, and 0.1 °C/s to 4 °C. The A/B adapter mix was then diluted from 5 to 0.33 μM by adding 1X T4 DNA Ligase Buffer (New England BioLabs) to the A/B adapter mix. The A/B adapter mix was then placed on ice, and 20 μL of the 0.33 μM A/B adapter mix was added to each DNA sample. A ligase mix was then added to each sample (Additional file 1: Table S12), and the samples were incubated at 22 °C for 4 h. The samples were then purified using a MinElute PCR purification kit (Qiagen) following the manufacturer’s instructions. Adapter-adapter ligation products were removed through RNACLEAN XP bead purification (Agencourt). The clear supernatant containing the RNACLEAN XP beads was used as PEG-wash Buffer. RNACLEAN XP beads were resuspended by vortexing, and 150 μL was added to each sample. The samples were incubated at 22 °C on a thermomixer with maximum shaking for “1 min on and 5 min off” for a total of 60 min. The samples were placed on a magnetic stand (Millipore), and the supernatant was removed. The beads were washed with the PEG-wash buffer, and the samples were incubated on a thermomixer at 22 °C for 2 min with maximum shaking. The samples were placed back on the magnetic stand, and the supernatant was removed. The samples were washed three times with 80 % ethanol while remaining on the magnetic stand, and the supernatant was discarded. The beads were then allowed to dry by room temperature incubation. DNA was eluted off the beads by adding TE buffer and alternating 2 min incubations at 42 °C and 5 s vortexes for a total of 8 min. The samples were placed back on the magnetic stand, and the DNA containing supernatant was transferred to a new tube. The samples were then purified using a MinElute PCR purification kit (Qiagen) following the manufacturer’s instructions. PCR reactions for multiplexed sequencing were then set up by adding a forward primer mix to each sample (Additional file 1: Table S13). The samples were incubated on ice for 5 min. Phusion Hot Start Flex DNA Polymerase 1 μL (New England BioLabs) and a designated reverse PCR primer 5 μL were added to each sample. The samples were then incubated in a PCR machine with the following settings: 80 °C for 2 min, 98 °C for 1 min, 2 cycles [98 °C for 30 s, 60 °C for 30 s, 72 °C for 30 s], 13 cycles [98 °C for 30 s, 66 °C for 30 s, 72 °C for 30 s], 4 cycles [98 °C for 30 s, 66 °C for 30 s, 72 °C for 60 s], and 72 °C for 7 min and then 4 °C. The samples were then purified using a Qiaquick PCR purification kit (Qiagen) following the manufacturer’s instructions. The samples were then pooled in equimolar concentrations and single-end ChIP-seq (50 nt) was performed on an Illumina HiSeq machine, resulting in 10–20 million reads/sample. The quality of samples was assessed using FastQC (http://www.bioinformatics.babraham.ac.uk/projects/fastqc/) and ChIPQC [44]. All samples passed quality control measures, which are outlined in Additional file 1: Table S14.

### ChIP-seq mapping and data analysis

This study makes use of data generated by the Blueprint Consortium. A full list of the investigators who contributed to the generation of the data is available from http://www.blueprint-epigenome.eu. Funding for the project was provided by the European Union’s Seventh Framework Programme (FP7/2007-2013) under grant agreement no. 282510—BLUEPRINT. The Blueprint dataset utilized was EGAD00001000913, which included ChIP-seq for H3K9me3 (2 μg antibody, Diagenode), H3K27me3 (1 μg antibody, Diagenode), H3K4me1 (2 μg antibody, Diagenode), H3K27ac (1 μg antibody, Diagenode), and H3K36me3 (5 μg antibody, Diagenode) on CD14-positive, CD16-negative classical monocytes from term neonatal umbilical cord blood and adult peripheral blood. For each of the histone modifications, three term neonatal samples and three adult samples were analyzed from the raw reads. Adapter sequences were trimmed from raw reads using Scythe (https://github.com/vsbuffalo/scythe), and the remaining reads were aligned to the Homo Sapiens genome assembly (Build 37, hg19) using Bowtie2 version 2.0.0 with default parameters [45]. Results were normalized to total read count. Peak calling was performed by comparing the ChIP samples to input samples with MACS2 [46]. Consensus peaksets were derived from the biological replicates using DiffBind and peaks needed to be present in two out of three replicates to be included in the consensus peaksets [47]. These consensus peaksets were then used for downstream analyses. Differentially bound sites were identified, and differential analysis was performed using the EdgeR package [48]. Sites were only considered to be differentially bound if the false discovery rate was <0.1. ChIPpeakAnno [49] was used to annotate enriched peaks and identify Gene Ontology terms associated with adjacent genes.

### Statistical analysis

Prism 6 was used for basic data analysis. The differences between groups were evaluated with the student’s *t* test for parametric quantitative data, the Mann-Whitney test for nonparametric quantitative data, and ANOVA for multiple comparisons of parametric data. Values of *p* < 0.05 were considered to be significant.

## Additional files

Additional file 1: Table S1. Total Number and Location of Monocyte H3K4me3 Peaks by Age. Table S2. Immunologically Relevant Gene Ontology Pathways Enriched for H3K4me3 in Monocytes. Table S3. microRNA Promoters with Differentially Bound H3K4me3 Between Neonatal and Adult Monocytes. Table S4. Genes Vital to Glycolysis and Lipid Metabolism with Differentially Bound H3K4me3 Between Adult and Neonatal Monocytes. Table S5. Gene Promoters with Differentially Bound H3K4me3 Between Adult and Term Neonatal Monocytes. Table S6. ChIP Primers. Table S7. ChIP-seq Patient Sample Composition. Table S8. ChIP-seq Kinase Mix. Table S9. ChIP-seq Blunting Mix. Table S10. ChIP-seq dA-tailing Mix. Table S11. ChIP-seq Oligonucleotide Adapters, Forward and Reverse PCR Primers. Table S12. ChIP-seq Ligase Mix. Table S13. ChIP-seq Forward Primer Mix. Table S14. H3K4me3 ChIP-seq Quality Control Data. (DOCX 41 kb) Additional file 2: Figure S1. Gene ontology pathways associated with H3K4me3 peaks present only in adult monocytes. (A) Broad biological gene ontology pathways associated with H3K4me3 monocyte peaks present in adult monocytes only. (B) Top 20 non-synonymous biological gene ontology pathways associated with adult monocyte H3K4me3 peaks. (PDF 36 kb) Additional file 3: Figure S2. There are very few bivalent domains in neonatal and adult monocytes. (A) Venn diagram showing overlapping adult monocyte consensus peaks for H3K4me3, H3K9me3, and H3K27me3. (B) Venn diagram showing overlapping term neonatal monocyte consensus peaks for H3K4me3, H3K9me3, and H3K27me3. H3K4me3 consensus peaks are obtained after only peaks present in at least two replicates are combined and analyzed. (PDF 680 kb) Additional file 4: Figure S3. Adult monocytes demonstrate increased levels of the surface receptors CCR2 and CD300C by flow cytometry. (A) Representative density plot of CD14 + CCR2+ neonatal monocytes. (B) Representative density plot of CD14 + CCR2+ adult monocytes. (C) Representative density plot of CD14 + CD300C+ neonatal monocytes. (D) Representative density plot of CD14 + CD300C+ adult monocytes. (E) Representative density plot of CD14 + CXCR4+ neonatal monocytes. (F) Representative density plot of CD14 + CXCR4+ adult monocytes. (PDF 425 kb) Additional file 5: Figure S4. Differential binding of H3K4me3 at pro-inflammatory cytokine promoters during development results in differences in gene expression. (A) Increased binding of H3K4me3 at the promoter site of *IL1B* in adult monocytes is associated with increased *IL1B* mRNA and protein expression after LPS stimulation. (B) Increased binding of H3K4me3 at the promoter site of *TNF* in adult monocytes is associated with increased *TNF* mRNA and protein expression after LPS stimulation. A = adult, T = term. H3K4me3 peaks display the read coverage for H3K4me3 from one of the representative replicates from each group. Black bars display the H3K4me3 consensus peaks after only peaks present in at least two replicates are combined and analyzed. For the mRNA studies, adult *n* = 5, term *n* = 5. For the protein studies, adult *n* = 4, term *n* = 4. **p* < 0.05, ***p* < 0.01. (PDF 215 kb)

## Abbreviations

Adult: Healthy adult
ChIP: Chromatin immunoprecipitation
ChIP-seq: Chromatin immunoprecipitation followed by massively parallel sequencing
O30: 30–36 weeks gestation
Term: 37+ weeks gestation
TSSs: Transcriptional start sites
U30: Under 30 weeks gestation
