# Supplementary material for: Neonatal monocytes exhibit a unique histone modification landscape
Source: Clin Epigenetics. 2016 Sep 20;8:99. doi: 10.1186/s13148-016-0265-7 (PMC5028999; doi:10.1186/s13148-016-0265-7)
Supplement: Additional file 1: Table S1. — Total Number and Location of Monocyte H3K4me3 Peaks by Age. Table S2. Immunologically Relevant Gene Ontology Pathways Enriched for H3K4me3 in Monocytes. Table S3. microRNA Promoters with Differentially Bound H3K4me3 Between Neonatal and Adult Monocytes. Table S4. Genes Vital to Glycolysis and Lipid Metabolism with Differentially Bound H3K4me3 Between Adult and Neonatal Monocytes. Table S5. Gene Promoters with Differentially Bound H3K4me3 Between Adult and Term Neonatal Monocytes. Table S6. ChIP Primers. Table S7. ChIP-seq Patient Sample Composition. Table S8. ChIP-seq Kinase Mix. Table S9. ChIP-seq Blunting Mix. Table S10. ChIP-seq dA-tailing Mix. Table S11. ChIP-seq Oligonucleotide Adapters, Forward and Reverse PCR Primers. Table S12. ChIP-seq Ligase Mix. Table S13. ChIP-seq Forward Primer Mix. Table S14. H3K4me3 ChIP-seq Quality Control Data. (DOCX 41 kb) [file 13148_2016_265_MOESM1_ESM.docx]

**Table S1:** Total Number and Location of Monocyte H3K4me3 Peaks by Age

|  | U30 | O30 | Term | Adult |
| --- | --- | --- | --- | --- |
| Total Consensus Peaks | 832 | 590 | 6370 | 20765 |
| Promoter Peaks | 22 | 18 | 605 | 12501 |
| Exon Peaks | 18 | 9 | 325 | 2097 |
| Intron Peaks | 151 | 120 | 1121 | 2699 |
| Intergenic Peaks | 641 | 443 | 4319 | 3468 |

**Table S2:** Immunologically Relevant Gene Ontology Pathways Enriched for H3K4me3 in Monocytes

| Age Group | GO Term | Description | # of Genes | p-value |
| --- | --- | --- | --- | --- |
| O30 |  |  |  |  |
|  | GO:0050851 | Antigen receptor-mediated signaling pathway | 3 | .033 |
|  | GO:1990266 | Neutrophil migration | 2 | .038 |
|  | GO:0045059 | Positive thymic T cell selection | 1 | .046 |
|  | GO:0034162 | Toll-like receptor 9 signaling pathway | 2 | .048 |
| Term |  |  |  |  |
|  | GO:1903039 | Positive regulation of leukocyte cell-cell adhesion | 25 | .008 |
|  | GO:0035455 | Response to interferon-alpha | 5 | .008 |
|  | GO:0019079 | Viral genome replication | 14 | .008 |
|  | GO:0038093 | Fc receptor signaling pathway | 30 | .009 |
|  | GO:0045582 | Positive regulation of T cell differentiation | 11 | .010 |
|  | GO:1990266 | Neutrophil migration | 11 | .011 |
|  | GO:1902107 | Positive regulation of leukocyte differentiation | 17 | .012 |
|  | GO:0050870 | Positive regulation of T cell activation | 24 | .013 |
|  | GO:0045070 | Positive regulation of viral genome replication | 6 | .013 |
|  | GO:0031664 | Positive regulation of lipopolysaccharide-mediated signaling pathway | 5 | .013 |
|  | GO:0002768 | Immune response-regulating cell surface receptor signaling pathway | 40 | .014 |
|  | GO:0071636 | Positive regulation of transforming growth factor beta production | 4 | .015 |
|  | GO:0045069 | Regulation of viral genome replication | 11 | .016 |
|  | GO:0030593 | Neutrophil chemotaxis | 10 | .021 |
|  | GO:0002696 | Positive regulation of leukocyte activation | 30 | .024 |
|  | GO:0002429 | Immune response-activating cell surface receptor signaling pathway | 27 | .027 |
|  | GO:0097530 | Granulocyte migration | 12 | .029 |
|  | GO:0032743 | Positive regulation of interleukin-2 production | 6 | .030 |
|  | GO:0045621 | Positive regulation of lymphocyte differentiation | 11 | .031 |
|  | GO:0051251 | Positive regulation of lymphocyte activation | 27 | .037 |
|  | GO:0045059 | Positive thymic T cell selection | 3 | .037 |
|  | GO:0072606 | Interleukin-8 secretion | 4 | .039 |
|  | GO:0071621 | Granulocyte chemotaxis | 11 | .039 |
|  | GO:0071559 | Response to transforming growth factor beta stimulus | 25 | .041 |
|  | GO:0045580 | Regulation of T cell differentiation | 13 | .044 |
|  | GO:1903037 | Regulation of leukocyte cell-cell adhesion | 29 | .045 |
|  | GO:0002764 | Immune response-regulating signaling pathway | 49 | .045 |
|  | GO:0033089 | Positive regulation of T cell differentiation in thymus | 3 | .049 |
|  | GO:0046719 | Regulation by virus of viral protein levels in host cell | 3 | .049 |
|  | GO:0002757 | Immune response-activating signal transduction | 39 | .049 |
| Adult |  |  |  |  |
|  | GO:0016032 | Viral process | 263 | 9.1E-5 |
|  | GO:0017015 | Regulation of transforming growth factor beta receptor signaling pathway | 42 | .004 |
|  | GO:1903844 | Regulation of cellular response to transforming growth factor beta stimulus | 42 | .004 |
|  | GO:0034166 | Toll-like receptor 10 signaling pathway | 29 | .005 |
|  | GO:0002224 | Toll-like receptor signaling pathway | 56 | .006 |
|  | GO:0002756 | MyD88-independent toll-like receptor signaling pathway | 34 | .006 |
|  | GO:0034146 | Toll-like receptor 5 signaling pathway | 29 | .006 |
|  | GO:0032897 | Negative regulation of viral transcription | 13 | .008 |
|  | GO:0002218 | Activation of innate immune response | 68 | .009 |
|  | GO:0034142 | Toll-like receptor 4 signaling pathway | 43 | .009 |
|  | GO:0019080 | Viral gene expression | 73 | .009 |
|  | GO:0034138 | Toll-like receptor 3 signaling pathway | 35 | .009 |
|  | GO:0034162 | Toll-like receptor 9 signaling pathway | 31 | .011 |
|  | GO:0035666 | TRIF-dependent toll-like receptor signaling pathway | 32 | .011 |
|  | GO:0045089 | Positive regulation of innate immune response | 80 | .011 |
|  | GO:0002758 | Innate immune response-activating signal transduction | 65 | .012 |
|  | GO:0030512 | Negative regulation of transforming growth factor beta receptor signaling pathway | 30 | .013 |
|  | GO:1903845 | Negative regulation of cellular response to transforming growth factor beta stimulus | 30 | .013 |
|  | GO:0046782 | Regulation of viral transcription | 32 | .013 |
|  | GO:0006924 | Activation-induced cell death of T cells | 7 | .017 |
|  | GO:0038123 | Toll-like receptor TLR1:TLR2 signaling pathway | 29 | .019 |
|  | GO:0038124 | Toll-like receptor TLR6:TLR2 signaling pathway | 29 | .019 |
|  | GO:0002221 | Pattern recognition receptor signaling pathway | 63 | .020 |
|  | GO:1903902 | Positive regulation of viral life cycle | 37 | .021 |
|  | GO:0045824 | Negative regulation of innate immune response | 15 | .022 |
|  | GO:0002755 | MyD88-dependent toll-like receptor signaling pathway | 33 | .024 |
|  | GO:0060761 | Negative regulation of response to cytokine stimulus | 17 | .026 |
|  | GO:0019083 | Viral transcription | 67 | .027 |
|  | GO:0042590 | Antigen processing and presentation of exogenous peptide antigen via MHC class I | 31 | .029 |
|  | GO:0007179 | Transforming growth factor beta receptor signaling pathway | 67 | .030 |
|  | GO:0002921 | Negative regulation of humoral immune response | 7 | .031 |
|  | GO:0043922 | Negative regulation by host of viral transcription | 7 | .031 |
|  | GO:0050687 | Negative regulation of defense response to virus | 9 | .031 |
|  | GO:0034121 | Regulation of toll-like receptor signaling pathway | 16 | .032 |
|  | GO:0034134 | Toll-like receptor 2 signaling pathway | 30 | .034 |
|  | GO:0045088 | Regulation of innate immune response | 96 | .036 |
|  | GO:0061515 | Myeloid cell development | 22 | .039 |
|  | GO:0060333 | Interferon-gamma-mediated signaling pathway | 33 | .041 |
|  | GO:0002479 | Antigen processing and presentation of exogenous peptide antigen via MHC class I, TAP-dependent | 29 | .041 |
|  | GO:0048524 | Positive regulation of viral process | 37 | .046 |
|  | GO:1903900 | Regulation of viral life cycle | 60 | .047 |

**Table S3:** microRNA Promoters with Differentially Bound H3K4me3 Between Neonatal and Adult Monocytes

| microRNA | p-value | FDR^1^ |
| --- | --- | --- |
| miR-1199 | 3.90E-05 | 0.001 |
| miR-1244 | 0.0003 | 0.006 |
| miR-1252 | 0.0003 | 0.006 |
| miR-1908 | 0.001 | 0.018 |
| miR-21 | 0.0006 | 0.010 |
| miR-3652 | 0.004 | 0.039 |
| miR-3658 | 0.002 | 0.027 |
| miR-4691 | 0.001 | 0.021 |
| miR-572 | 0.0008 | 0.012 |
| miR-620 | 0.0001 | 0.002 |
| miR-6516 | 4.82E-05 | 0.001 |
| miR-652 | 0.003 | 0.031 |
| miR-661 | 8.19E-06 | 0.0003 |
| miR-663AHG | 0.0003 | 0.006 |
| miR-664b | 0.003 | 0.033 |
| miR-6748 | 0.0004 | 0.008 |
| miR-6758 | 0.003 | 0.030 |
| miR-6836 | 0.001 | 0.015 |
| miR-7110 | 6.63E-05 | 0.001 |
| miR-769 | 4.94E-09 | 7.82E-07 |

^1^FDR=false discovery rate. Only genes with FDR <0.05 were included.

**Table S4:** Genes Vital to Glycolysis and Lipid Metabolism with Differentially Bound H3K4me3 Between Adult and Neonatal Monocytes

| Metabolic Pathway | Gene | p-value | FDR^1^ |
| --- | --- | --- | --- |
| Glycolysis |  |  |  |
|  | *PFKP* | 3.22E-06 | 0.0001 |
|  | *AKT1* | 0.005 | 0.047 |
| Lipid Metabolism |  |  |  |
|  | *ACSL1* | 4.24E-06 | 0.0002 |
|  | *ABHD12* | 6.66E-06 | 0.0002 |
|  | *ACADVL* | 6.83E-06 | 0.0002 |
|  | *ABHD6* | 2.18E-05 | 0.0007 |
|  | *ACSF3* | 0.0019 | 0.022 |
|  | *AGPAT3* | 0.003 | 0.03 |

^1^FDR=false discovery rate. Only genes with FDR <0.05 were included.

**Table S5:** Gene Promoters with Differentially Bound H3K4me3 Between Adult and Term Neonatal Monocytes

| Gene | p-value | FDR^1^ |
| --- | --- | --- |
| *VCAN* | 5.54E-34 | 2.39E-30 |
| *ADGRG3* | 3.48E-22 | 6.82E-19 |
| *FGD2* | 8.09E-21 | 1.45E-17 |
| *KREMEN1* | 2.42E-20 | 3.73E-17 |
| *GP1BB* | 1.25E-19 | 1.79E-16 |
| *TBC1D9* | 2.07E-18 | 2.35E-15 |
| *DOK2* | 1.31E-17 | 1.42E-14 |
| *MYCL* | 2.01E-17 | 2.06E-14 |
| *GIMAP7* | 5.94E-17 | 5.12E-14 |
| *FZD1* | 2.03E-16 | 1.62E-13 |
| *CD68* | 2.11E-16 | 1.62E-13 |
| *OAF* | 9.35E-16 | 6.71E-13 |
| *PLXNB2* | 2.43E-15 | 1.64E-12 |
| *ANXA2* | 3.75E-15 | 2.37E-12 |
| *PPARGC1B* | 1.25E-14 | 6.75E-12 |
| *IRF8* | 2.64E-14 | 1.30E-11 |
| *LRP1* | 2.66E-14 | 1.30E-11 |
| *SH3TC1* | 1.84E-13 | 8.45E-11 |
| *PRKAG2* | 3.23E-13 | 1.39E-10 |
| *MDFIC* | 5.46E-13 | 2.22E-10 |
| *NRROS* | 2.06E-12 | 7.79E-10 |
| *FRMD4B* | 3.04E-12 | 1.13E-09 |
| *CXXC5* | 3.76E-12 | 1.37E-09 |
| *VAV2* | 4.23E-12 | 1.52E-09 |
| *QPRT* | 4.58E-12 | 1.62E-09 |
| *UHRF1BP1* | 6.10E-12 | 2.02E-09 |
| *INHBB* | 7.75E-12 | 2.49E-09 |
| *PHOSPHO1* | 8.48E-12 | 2.69E-09 |
| *SLC12A7* | 8.79E-12 | 2.74E-09 |
| *PRKCH* | 1.02E-11 | 3.12E-09 |
| *PIK3IP1* | 1.13E-11 | 3.38E-09 |
| *ZC3H12C* | 1.46E-11 | 4.30E-09 |
| *DYRK2* | 2.58E-11 | 7.32E-09 |
| *IL15* | 3.66E-11 | 9.98E-09 |
| *RUSC2* | 5.83E-11 | 1.53E-08 |
| *CCDC109B* | 1.32E-10 | 3.23E-08 |
| *TBC1D12* | 1.69E-10 | 4.09E-08 |
| *CYP1B1-AS1* | 2.13E-10 | 5.10E-08 |
| *FAM78A* | 2.53E-10 | 5.93E-08 |
| *KCNA3* | 2.80E-10 | 6.41E-08 |
| *SOCS6* | 2.99E-10 | 6.70E-08 |
| *RTN1* | 5.60E-10 | 1.21E-07 |
| *PCSK5* | 7.76E-10 | 1.59E-07 |
| *ICA1L* | 9.38E-10 | 1.89E-07 |
| *ZNF507* | 1.01E-09 | 2.02E-07 |
| *SLC2A6* | 1.91E-09 | 3.52E-07 |
| *MITF* | 2.17E-09 | 3.93E-07 |
| *CD33* | 2.42E-09 | 4.30E-07 |
| *EPHB4* | 2.63E-09 | 4.64E-07 |
| *CYB5RL* | 3.60E-09 | 6.10E-07 |

^1^FDR=false discovery rate. Only genes with FDR <0.05 were included.

**Table S6:** ChIP Primers

| Promoter | Orientation | Primer Sequence |
| --- | --- | --- |
| *IL1B* | Sense  Antisense | 5’ –GAGGCTATCCAGATGTGTTGTTGC- 3’  5’ –GTCTTTTCTCCCCATTTGCCTCTG- 3’ |
| *IL6* | Sense  Antisense | 5’ – CCAACGGGGCCGACTAGACTGACT- 3’  5’ – GGAAGCCCTGAGAAGCAATAACC- 3’ |
| *IL12B* | Sense  Antisense | 5’ –GGGGGTGCCTTGAGTGTGGTTGT- 3’  5’ –ATAGGGGCGGATGGGAGCAGAA- 3’ |
| *TNF* | Sense  Antisense | 5’ –CTGAGGGGAGGGAGGAGGGAAGTC- 3’  5’ –GAAACACCCCCGAGCAATCCAGT- 3’ |

**Table S7:** ChIP-seq Patient Sample Composition

| Group | Replicate | Ages | # of Cells used in ChIP |
| --- | --- | --- | --- |
| U30 | 1 | 24, 24.86, 29.14 (weeks gestation) | 8.8x10^5^ |
| U30 | 2 | 28.14, 29.14, 29.29 (weeks gestation) | 8.5x10^5^ |
| U30 | 3 | 28.14, 28.14, 29.29 (weeks gestation) | 8.2x10^5^ |
| O30 | 1 | 30.71, 31.43, 31.43 (weeks gestation) | 7.0x10^5^ |
| O30 | 2 | 32.43, 33.43, 34.29 (weeks gestation) | 5.5x10^5^ |
| O30 | 3 | 31.71, 33.43, 34 (weeks gestation) | 6.0x10^5^ |
| Term | 1 | 37.14, 38, 41 (weeks gestation) | 5.0x10^5^ |
| Term | 2 | 39.86, 41, 41.14 (weeks gestation) | 5.5x10^5^ |
| Term | 3 | 39.43, 40.43, 41.14 (weeks gestation) | 6.0x10^5^ |
| Adult | 1 | 33 years | 2.1x10^6^ |
| Adult | 2 | 24 years | 2.7x10^6^ |
| Adult | 3 | 44 years | 1.4x10^6^ |

U30 = under 30 weeks gestation, O30 = 30-36 weeks gestation, Term = term gestation

**Table S8:** ChIP-seq Kinase Mix

| Reagent | Amount (microliters) | Manufacturer |
| --- | --- | --- |
| T4 Polynucleotide Kinase | 1.5 | New England BioLabs |
| 10X NEBNext End Repair Reaction Buffer | 19 | New England BioLabs |
| Total added to each sample | 20.5 |  |

**Table S9:** ChIP-seq Blunting Mix

| Reagent | Amount (microliters) | Manufacturer |
| --- | --- | --- |
| Buffer 2 | 12 | New England BioLabs |
| Bovine Serum Albumin | 1.2 | New England BioLabs |
| Water | 12 |  |
| T4 DNA Polymerase | 0.3 | New England BioLabs |
| Total added to each sample | 25.5 |  |

**Table S10:** ChIP-seq dA-tailing Mix

| Reagent | Amount (microliters) | Manufacturer |
| --- | --- | --- |
| 10X NEBNext dA Tailing Buffer | 11 | New England BioLabs |
| Water | 18 |  |
| Klenow Fragment (3’ -> 5’ exo-) | 1 | New England BioLabs |
| Total added to each sample | 30 |  |

**Table S11:** ChIP-seq Oligonucleotide Adapters, Forward and Reverse PCR Primers

| Oligonucleotide | Sequence (5’ to 3’) |
| --- | --- |
| Adapters |  |
| Adapter A | CTCTTTCCCTACACGACGCTCTTCCGATC*T |
| Adapter B | /5Phos/GATCGGAAGAGCACACGTCTGAAC*TCC/3C6/ |
| Universal Forward Primer | AATGATACGGCGACCACCGAGATCTACACTCTTTCCCTACACGACGCTCTTCCGATC*T |
| Reverse Primers for Multiplexing |  |
| R5 | CAAGCAGAAGACGGCATACGAGATCACTGTGTGACTGGAGTTCAGACGTGTGCTCTTCCGATC*T |
| R6 | CAAGCAGAAGACGGCATACGAGATATTGGCGTGACTGGAGTTCAGACGTGTGCTCTTCCGATC*T |
| R12 | CAAGCAGAAGACGGCATACGAGATTACAAGGTGACTGGAGTTCAGACGTGTGCTCTTCCGATC*T |
| R19 | CAAGCAGAAGACGGCATACGAGATTTTCACGTGACTGGAGTTCAGACGTGTGCTCTTCCGATC*T |

**Table S12:** ChIP-seq Ligase Mix

| Reagent | Amount (microliters) | Manufacturer |
| --- | --- | --- |
| 10X T4 DNA Ligase Buffer | 2 | New England BioLabs |
| Quick T4 DNA Ligase | 2 | New England BioLabs |
| 2X Quick Ligase Buffer | 90 | New England BioLabs |
| Total added to each sample | 94 |  |

**Table S13:** ChIP-seq Forward Primer Mix

| Reagent | Amount (microliters) | Manufacturer |
| --- | --- | --- |
| 25 uM Forward PCR Primer | 2 |  |
| 5X GC Phusion Buffer | 20 | New England BioLabs |
| dNTPs | 2.5 | New England BioLabs |
| 10X PCRx Enhancer Solution | 10 | Invitrogen |
| Water | 9.5 |  |
| Total added to each sample | 44 |  |

**Table S14:** H3K4me3 ChIP-seq Quality Control Data

| Sample | Sequencing Depth (reads) | Read Quality (Phred Score^1^) | Uniquely Mapping Reads | Reads Mapped (%) | SSD^2^ | FRIP^3^ |
| --- | --- | --- | --- | --- | --- | --- |
| U30G1 | 57,276,495 | 39 | 72,464 | 100 | 14.3 | 22.8 |
| U30G2 | 49,342,352 | 39 | 72,665 | 100 | 11.6 | 18.7 |
| U30G3 | 29,802,440 | 39 | 291,617 | 100 | 4.07 | 5.89 |
| O30G1 | 39,347,378 | 39 | 87,324 | 100 | 7.98 | 12 |
| O30G2 | 62,638,230 | 39 | 310,426 | 100 | 6.87 | 7.01 |
| O30G3 | 44,318,249 | 39 | 215,790 | 100 | 5.52 | 6.55 |
| TermG1 | 71,263,489 | 39 | 252,327 | 100 | 12.8 | 14.8 |
| TermG2 | 72,201,221 | 39 | 251,160 | 100 | 11.6 | 14 |
| TermG3 | 60,628,889 | 39 | 145,300 | 100 | 14.2 | 18.9 |
| AdultG1 | 82,180,974 | 39 | 376,983 | 100 | 10.1 | 32.4 |
| AdultG2 | 77,694,801 | 39 | 1,182,414 | 100 | 6.27 | 10.1 |
| AdultG3 | 77,679,834 | 39 | 400,871 | 100 | 10.8 | 31.7 |

^1^Phred scores greater than 30 are considered excellent, with a base call accuracy of over 99.9%.

^2^SSD is the standard deviation of signal pile-up along the genome normalized to the total number of reads; values greater than 2 indicate very good chromatin immunoprecipitation enrichment.

^3^FRIP is the percentage of reads that overlap peaks; values greater than 5 indicate successful chromatin immunoprecipitation enrichment.
