# Supplementary figures and images for: Neonatal monocytes exhibit a unique histone modification landscape
Source: Clin Epigenetics. 2016 Sep 20;8:99. doi: 10.1186/s13148-016-0265-7 (PMC5028999; doi:10.1186/s13148-016-0265-7)

A.

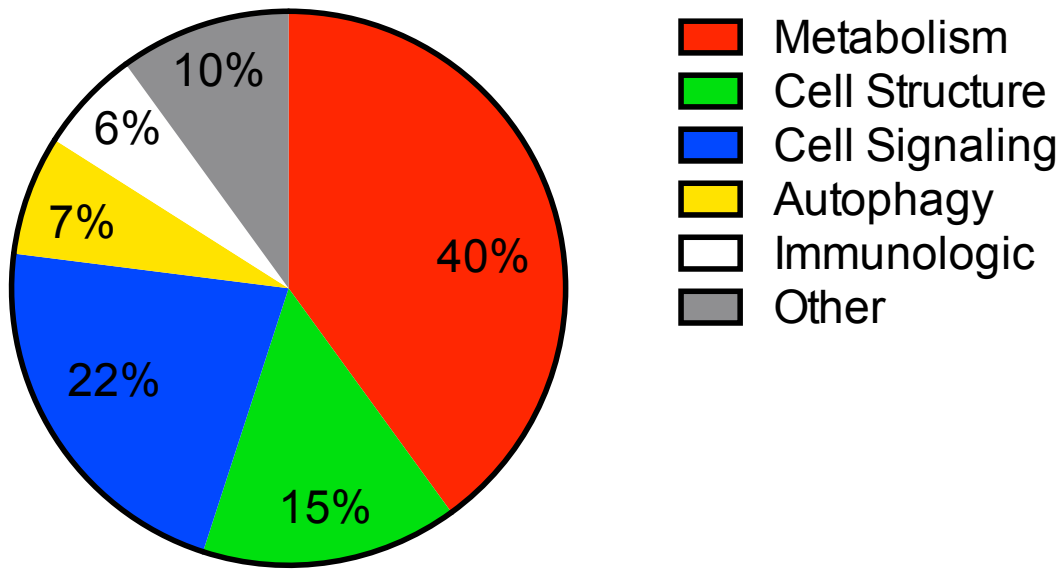

B.

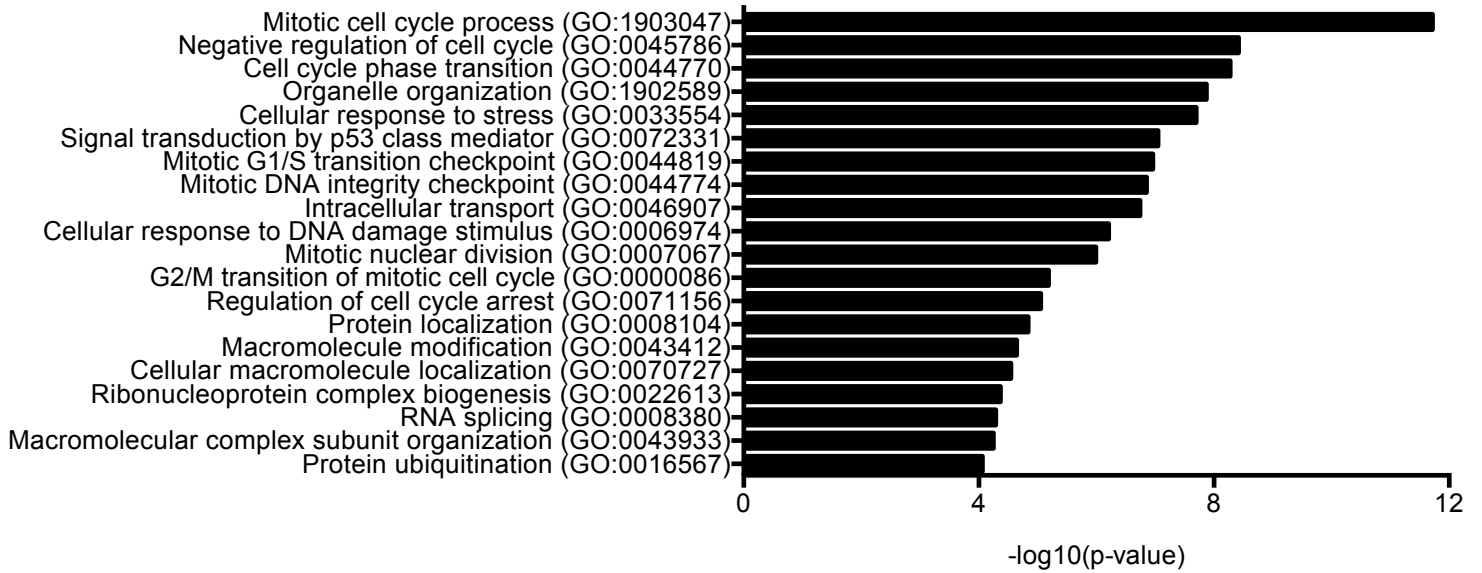

Supplement: Additional file 2: Figure S1. — Gene ontology pathways associated with H3K4me3 peaks present only in adult monocytes. (A) Broad biological gene ontology pathways associated with H3K4me3 monocyte peaks present in adult monocytes only. (B) Top 20 non-synonymous biological gene ontology pathways associated with adult monocyte H3K4me3 peaks. (PDF 36 kb) [file 13148_2016_265_MOESM2_ESM.pdf]

**A.**

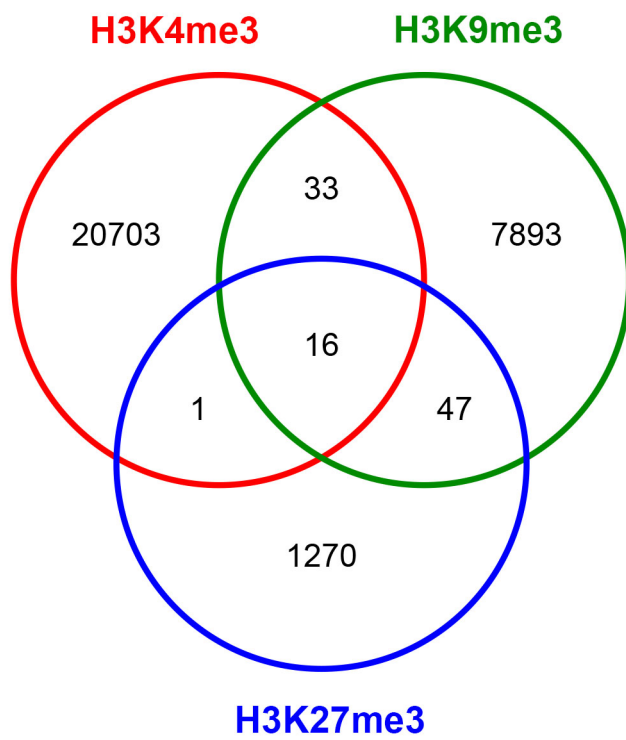

**B.**

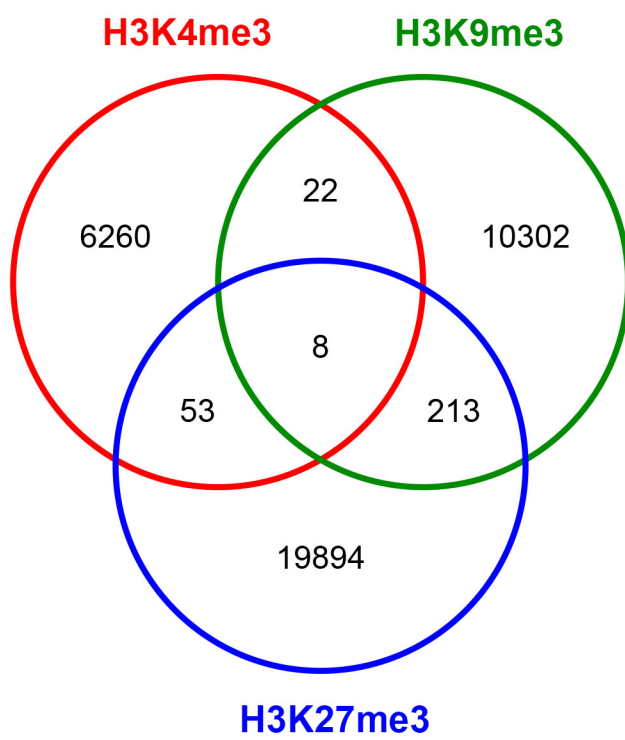

Supplement: Additional file 3: Figure S2. — There are very few bivalent domains in neonatal and adult monocytes. (A) Venn diagram showing overlapping adult monocyte consensus peaks for H3K4me3, H3K9me3, and H3K27me3. (B) Venn diagram showing overlapping term neonatal monocyte consensus peaks for H3K4me3, H3K9me3, and H3K27me3. H3K4me3 consensus peaks are obtained after only peaks present in at least two replicates are combined and analyzed. (PDF 680 kb) [file 13148_2016_265_MOESM3_ESM.pdf]

A.

Term Neonate

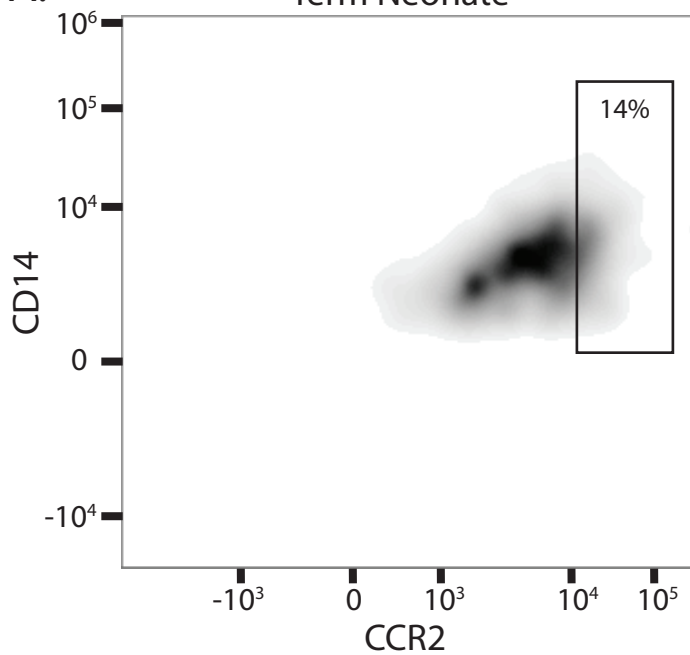

B.

Adult

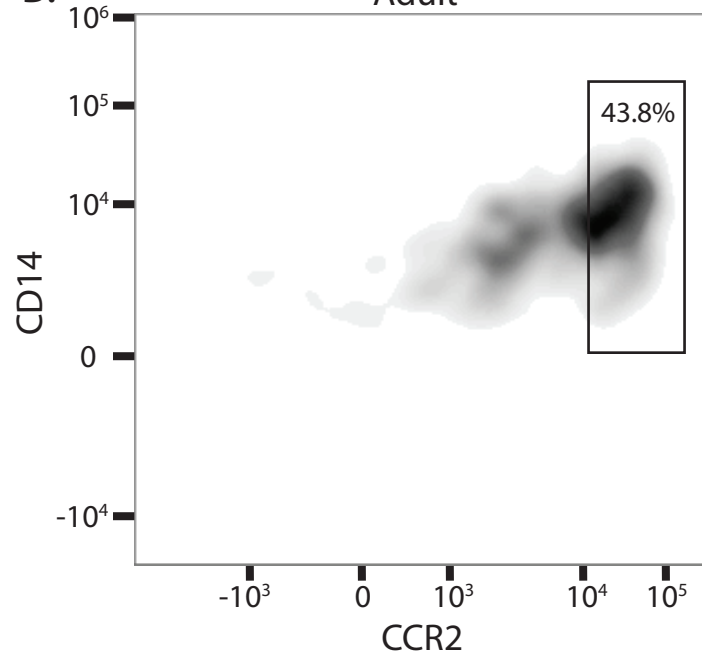

C.

Term Neonate

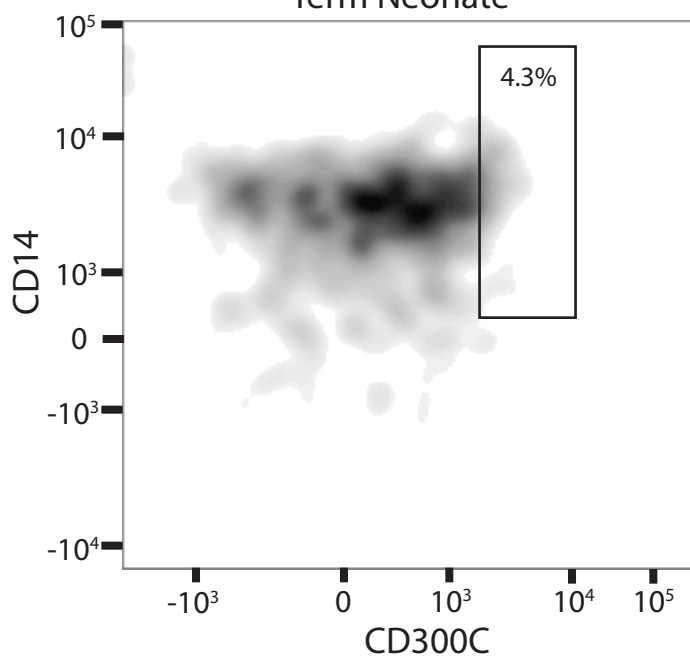

D.

Adult

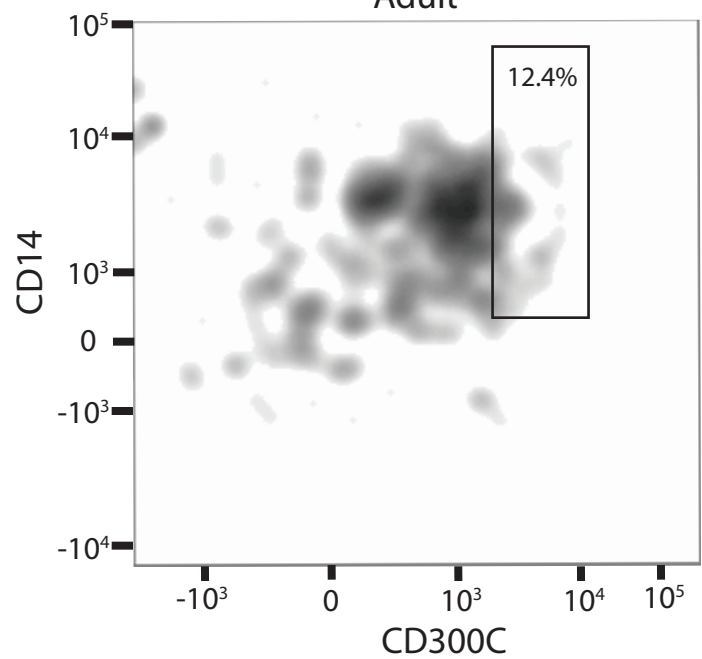

E.

Term Neonate

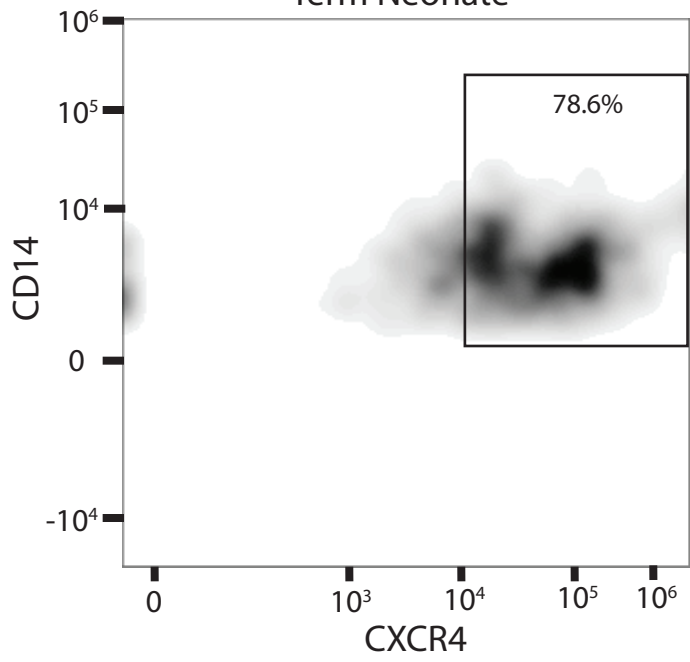

F.

Adult

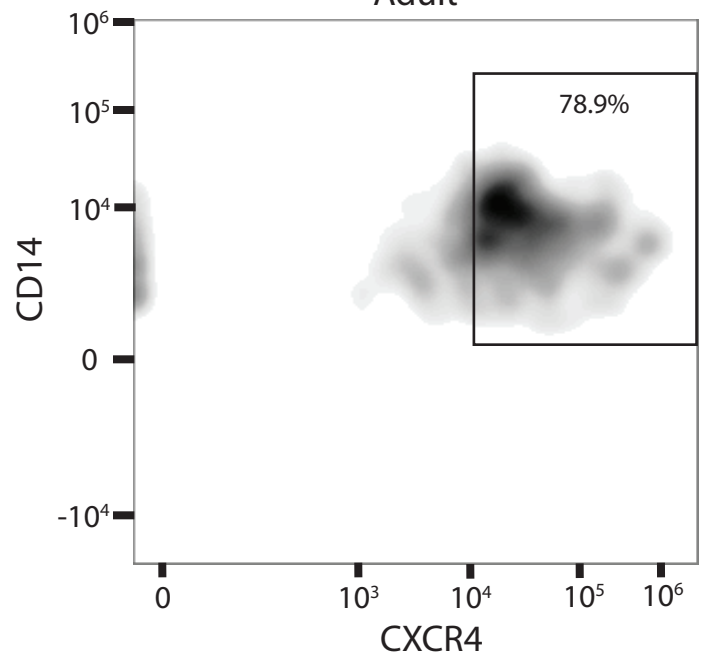

Supplement: Additional file 4: Figure S3. — Adult monocytes demonstrate increased levels of the surface receptors CCR2 and CD300C by flow cytometry. (A) Representative density plot of CD14 + CCR2+ neonatal monocytes. (B) Representative density plot of CD14 + CCR2+ adult monocytes. (C) Representative density plot of CD14 + CD300C+ neonatal monocytes. (D) Representative density plot of CD14 + CD300C+ adult monocytes. (E) Representative density plot of CD14 + CXCR4+ neonatal monocytes. (F) Representative density plot of CD14 + CXCR4+ adult monocytes. (PDF 425 kb) [file 13148_2016_265_MOESM4_ESM.pdf]

A.

*IL1B*

Differentially  
Bound

A H3K4me3

T H3K4me3

Gene

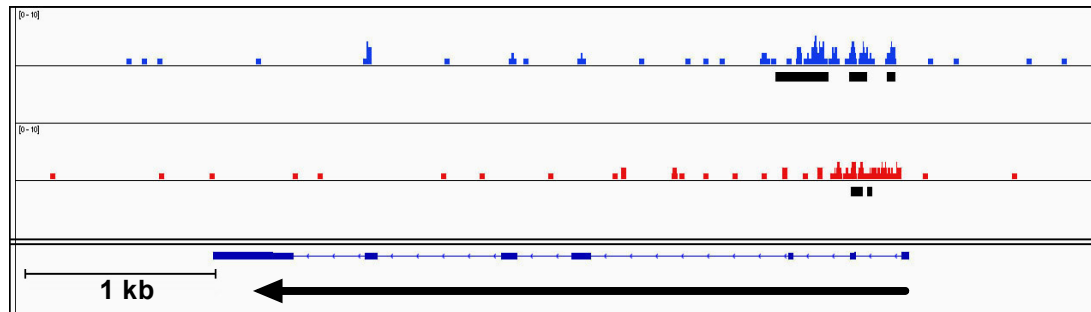

$p=1.1e^{-7}$

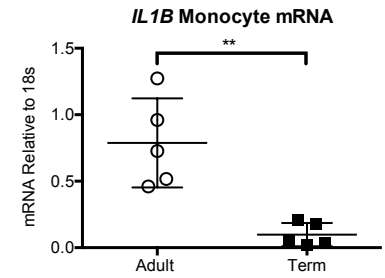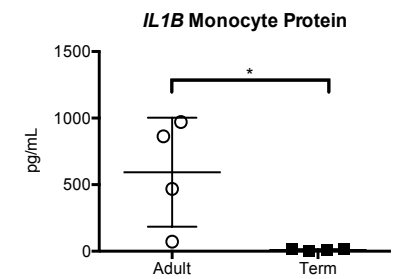

B.

*TNF*

A H3K4me3

T H3K4me3

Gene

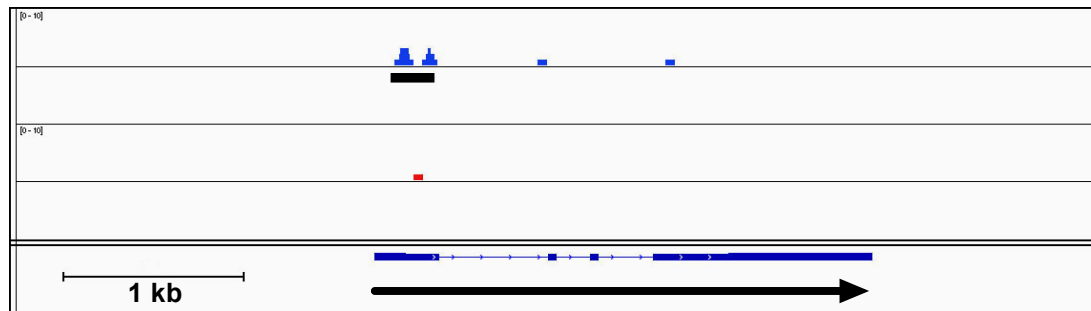

$p<0.05$

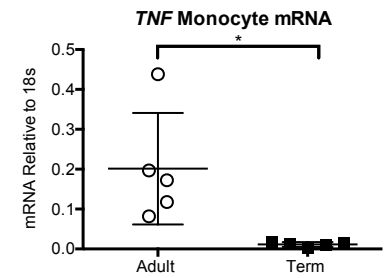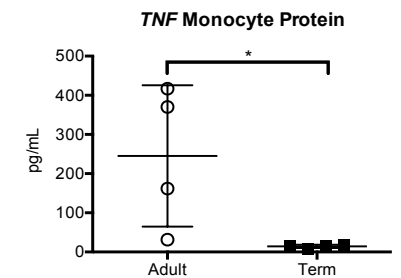

Supplement: Additional file 5: Figure S4. — Differential binding of H3K4me3 at pro-inflammatory cytokine promoters during development results in differences in gene expression. (A) Increased binding of H3K4me3 at the promoter site of IL1B in adult monocytes is associated with increased IL1B mRNA and protein expression after LPS stimulation. (B) Increased binding of H3K4me3 at the promoter site of TNF in adult monocytes is associated with increased TNF mRNA and protein expression after LPS stimulation. A = adult, T = term. H3K4me3 peaks display the read coverage for H3K4me3 from one of the representative replicates from each group. Black bars display the H3K4me3 consensus peaks after only peaks present in at least two replicates are combined and analyzed. For the mRNA studies, adult n = 5, term n = 5. For the protein studies, adult n = 4, term n = 4. *p < 0.05, **p < 0.01. (PDF 215 kb) [file 13148_2016_265_MOESM5_ESM.pdf]
